# Estrogen-activated MDM2 disrupts mammary tissue architecture through a p53-independent pathway

**DOI:** 10.18632/oncotarget.18147

**Published:** 2017-05-24

**Authors:** Nandini Kundu, Angelika Brekman, Jun Yeob Kim, Gu Xiao, Chong Gao, Jill Bargonetti

**Affiliations:** ^1^ The Department of Biological Sciences Hunter College, City University of New York, New York, NY 10065, USA; ^2^ PhD Program in Biology, The Graduate Center, City University of New York, New York, NY 10016, USA; ^3^ PhD Program in Biochemistry, The Graduate Center, City University of New York, New York, NY 10016, USA; ^4^ Department of Cell and Developmental Biology, Weill Cornell Medical College of Cornell University, New York, NY 10065, USA

**Keywords:** p53, MDM2, estrogen receptor, Rb, E2F1

## Abstract

The Cancer Genome Atlas (TCGA) data indicate that high MDM2 expression correlates with all subtypes of breast cancer. Overexpression of MDM2 drives breast oncogenesis in the presence of wild-type or mutant p53 (mtp53). Importantly, estrogen-receptor positive (ER+) breast cancers overexpress MDM2 and estrogen mediates this expression. We previously demonstrated that this estrogen-MDM2 axis activates the proliferation of breast cancer cell lines T47D (mtp53 L194F) and MCF7 (wild-type p53) in a manner independent of increased degradation of wild-type p53 (ie, p53-independently). Herein we present data supporting the role of the estrogen-MDM2 axis in regulating cell proliferation and mammary tissue architecture of MCF7 and T47D cells in a p53-independent manner. Inducible shRNA mediated MDM2 knockdown inhibited colony formation in soft agar, decreased mass size and induced lumen formation in matrigel and also significantly reduced mitosis as seen by decreased phospho-histone H3 positive cells. The knockdown of MDM2 in both cell lines decreased Rb phosphorylation and the level of E2F1 protein. This signaling was through the estrogen receptor because fulvestrant (a selective estrogen receptor degrader) decreased MDM2 protein levels and decreased phosphorylation of Rb. Taken together these data indicate that in some ER+ breast cancers the estrogen-MDM2-Rb-E2F1 axis is a central hub for estrogen-mediated p53-independent signal transduction. This is the first indication that estrogen signaling utilizes the estrogen-MDM2 axis to provoke phosphorylation of Rb and increase E2F1 while promoting abnormal mammary architecture.

## INTRODUCTION

A majority of breast cancers are estrogen–receptor alpha positive (ER+) and many of these are resistant to therapies targeting their hormone receptor status [1]. Many ER+ breast cancers have MDM2 overexpression suggesting that MDM2 is an ER+ axis driver oncogene that can be targeted for cancer therapy [2–4]. MDM2 overexpression is found in many different types of cancers [5, 6]. Some cancer patients with MDM2 overexpression have a worse prognosis than patients with cancers that do not have such overexpression [5]. Therefore it is not surprising that MDM2 expression is a negative prognostic marker for breast cancer [7]. ER+ cells exposed to estrogen show an increase in their transcription of *mdm2* and increased MDM2 protein [8–11]. The *mdm2* gene contains a single nucleotide polymorphism, SNP 309, where T is changed to G thereby further increasing estrogen activated MDM2 expression by increased recruitment of the Sp1 transcription factor to the promoter region of the gene [9, 12]. This *mdm2* SNP309 G allele accelerates tumor formation in a gender specific and hormone dependent manner [9]. While estrogen signaling up-regulates MDM2, it has not been determined if MDM2 is required for the estrogen-mediated phenotypic changes that are associated with tumorigenesis. Herein we investigate the estrogen-MDM2 axis as a pathway that functions in some ER+ cells to promote p53-independent tumorigenic outcomes.

There are significant pieces of evidence showing that MDM2 has strong cancer promoting properties that are independent of degrading p53 [11, 13–16]. Our previous work uncovered that estrogen signaling activates MDM2-mediated breast cancer proliferation in a p53-independent manner [11]. Most notably recent work has documented that estrogen receptor alpha mediates the p53-independent overexpression of both MDM2 and MDM4 in human breast cancers [13]. MDM2 expression in p53-null mice alters the tumor spectrum and rapidly promotes tumor formation [17]. Moreover forced MDM2 overexpression in both wild-type and p53-null mammary glands promotes cell cycle progression into S phase [18]. Particularly, proliferative targets of MDM2 include the stimulation of E2F1 transcriptional activity [19], increased E2F1 protein stability [20], disruption of the Rb-E2F1 complex and inhibition of tumor suppressive functions of Rb [21–23]. Estrogen treatment induces cell cycle progression through promoting the G1 to S phase transition [24] and promotes Rb phosphorylation [25]. This is also seen in prostate cancer cells where MDM2 depletion reduces E2F1 and phosphorylated Rb [26]. However, the connection between estrogen signaling, MDM2, and the Rb-E2F1 pathway has never been shown. We hypothesized that estrogen signaling mediated the disruption of mammary tissue architecture, and increased proliferative outcomes, through an MDM2-Rb-E2F1 axis.

One of the characteristics of malignancy is the disruption of normal mammary tissue architecture in the specific context of the extracellular matrix [27]. The central aspect of the mammary architecture is the acinus, which forms terminal ductal lobular units. Each acinus has a hollow lumen lined by a single layer of epithelial cells. This *in vivo* glandular architecture is lost in 2D culture due to loss of *in vivo* cell-to-cell communication and interactions with extracellular matrix proteins. This structure can be recapitulated when normal mammary epithelial cells are grown in 3D laminin-rich matrigel. Malignant cells grown in matrigel display a disrupted architecture and form disorganized colonies with a loss of tissue polarity and filled lumen [27]. Herein we investigated if in 3D matrigel conditions the estrogen-MDM2 signaling axis in the MCF7 and T47D colonies contributed to the filled lumen phenotype. We observed that MDM2 was required for blocking hollow lumen formation. When MDM2 was knocked down in cells grown in matrigel, the colonies exhibited hollow lumen formation and reduced the number of cells that were phospho-histone H3 positive. Moreover, MDM2 knockdown in T47D and MCF7 cells grown with estrogen in 2D conditions showed decreased phosphorylation of Rb. The estrogen-MDM2-Rb-E2F1 axis was blocked in the presence of the estrogen receptor antagonist fulvestrant. We show for the first time that at least in some cases the estrogen-MDM2 axis participates in a pathway that disrupts mammary tissue architecture, does not target p53 for degradation, and promotes activation of the E2F1 pathway.

## RESULTS

### MDM2 is required for estrogen to increase 3D colony size of estrogen receptor-positive breast cancer cells

**Figure 1:**
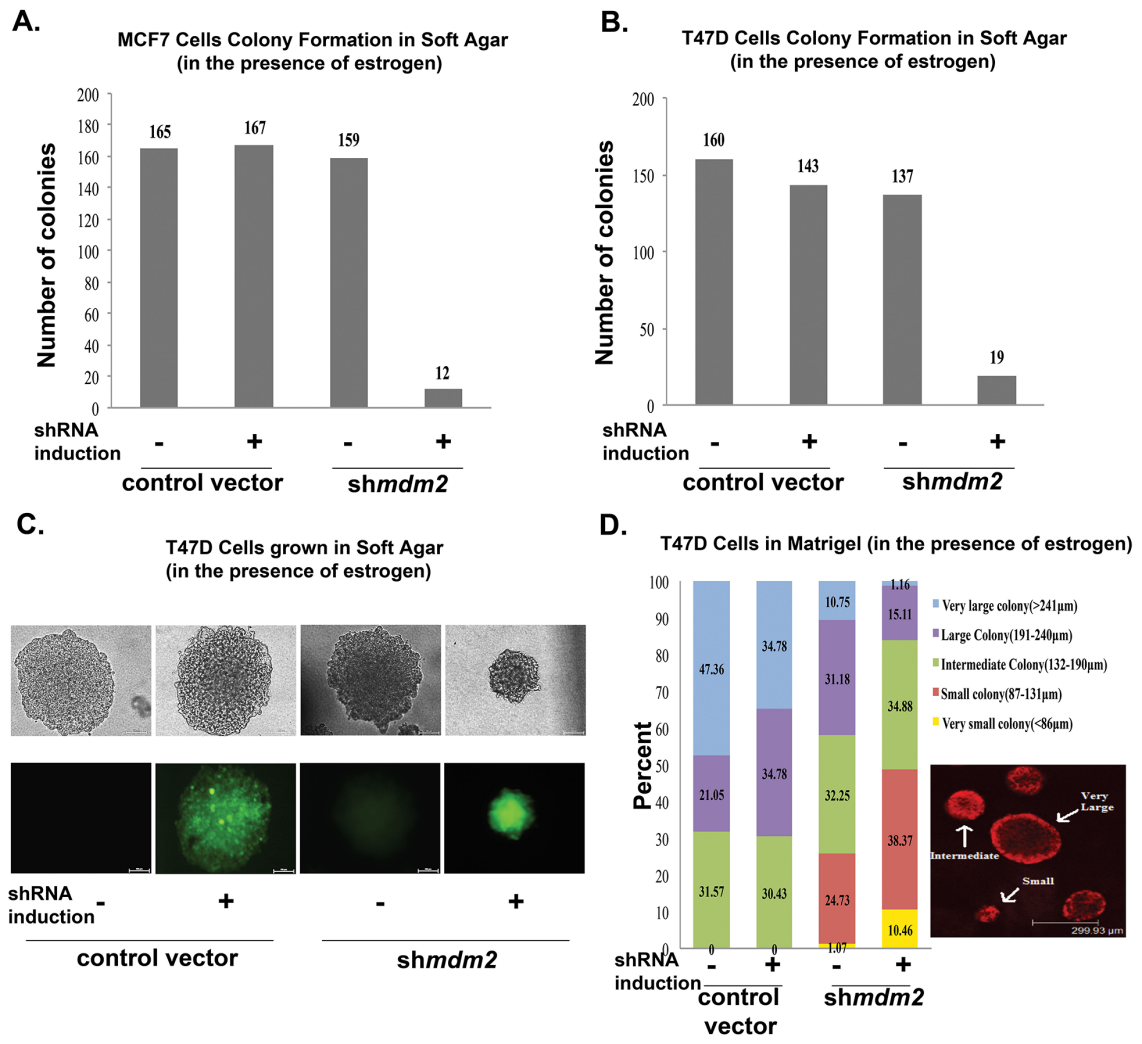
MDM2 depletion in estrogen-treated breast cancer cells inhibits colony formation in 3D culture conditions **(A)** Number of large colonies (50μm or larger) determined by counting the colonies of MCF7 cells when grown in soft agar in the presence of estrogen and in the presence or absence of shRNA induction (viewed by inverted fluorescence microscope). Average of three independent experiments are shown. The number of colonies for 3 independent experiments were as follows: control vector –shRNA induction (160, 158, 175), control vector + shRNA induction (153, 151, 197), *mdm2*shRNA –shRNA induction (140, 137, 200) and *mdm2*shRNA + shRNA induction (17, 11, 8). The p-value determined by 2-tailed Student t-test comparing with and without MDM2 knockdown was p-value=0.002. **(B)** Number of large colonies (100μm or larger) determined by counting the colonies of T47D cells when grown in soft agar in the presence of estrogen and in the presence or absence of shRNA (viewed by inverted fluorescence microscope). Average of three independent experiments are shown. The number of colonies for 3 independent experiments were as follows: control vector –shRNA induction (220, 191, 70), control vector + shRNA induction (202, 195, 33), *mdm2*shRNA –shRNA induction (166, 175, 69) and *mdm2*shRNA + shRNA induction (27,20, 9). The p-value determined by 2-tailed Student t-test comparing with and without MDM2 knockdown was p-value=0.02. **(C)** Representative images of colonies that T47D cells formed in soft agar in the presence or absence of MDM2 knockdown. **(D)** T47D cells grown in matrigel for 3 weeks in presence of estrogen and in presence or absence of 4μg/ml doxycycline, were fixed and stained with propidium iodide. Colony masses were categorized in 5 different groups and the number of masses in each group were counted and presented as percentages in the total population. This is an average of two independent experiments. The total number of masses scored for 2 independent experiments were control vector –shRNA induction (20, 15), control vector + shRNA induction (25, 27), *mdm2*shRNA –shRNA induction (93, 95) and *mdm2*shRNA + shRNA induction (86, 83). The p-value was determined by 2-tailed Student t-test. The p-value for large and small colonies for comparisons with and without MDM2 knockdown were p-value=0.03 and p-value=0.05 respectively. Two independent scorers counted the numbers of colonies for each independent experiment.

To determine if estrogen signaling for tumorigenic properties requires the p53-independent MDM2 pathway we evaluated the effect of MDM2 knockdown on estrogen driven MCF7 and T47D proliferation in soft agar. These ER+ breast cancer cell lines carry the *mdm2* SNP309 T to G change [9, 10, 12] and estrogen treatment induces their anchorage-independent cell growth in soft agar [28]. T47D cells express mtp53 L194F that does not activate transcription of p53 targets and also does not have oncogenic gain-of-function activity [29]. Estrogen treatment increases the p53-independent MDM2 signaling in T47D cell lines and also in MCF7 cells with wild-type p53 [10, 11]. To investigate how the estrogen-mediated increase in MDM2 protein affected anchorage independent growth of MCF7 and T47D cells we depleted MDM2 using a mir30-based shRNA targeting strategy (described in [11]). We evaluated MDM2 knockdown by western blots for cells grown in 2D culture as shown in Figures 4–6. Figures 4–6 are reflective of all MDM2 knockdown results. We observed a significant decrease in the size and number of colonies formed in soft agar with MDM2 knockdown (Figure 1A and 1B). Representative images of the colonies formed by T47D with and without shRNA induction are shown in Figure 1C. In order to test the influence of MDM2 on 3D culture colony size through an additional method we examined the growth of MCF7 and T47D cells in matrigel.

**Figure 2:**
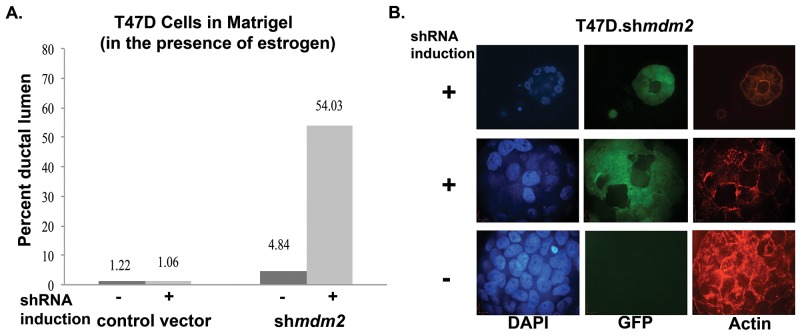
MDM2 depletion in ER+ breast cancer cells with mutant p53 leads to formation of lumen and reverts mammary architecture towards a normal state **(A)** T47D cells grown in matrigel for 3 weeks in presence of estrogen and in the presence or absence of 4 μg/ml dox, were fixed, stained with F-Actin and mounted with DAPI containing mounting media. Confocal z-stack images were acquired. Masses with lumen were counted and presented as percent of total number of masses grown in 3D matrigel. An average of two independent experiments are shown. The number of masses counted for 2 independent experiments were control vector -shRNA induction (21, 41), control vector +shRNA induction (21, 47), *mdm2*shRNA -shRNA induction (31, 46) and *mdm2*shRNA +shRNA induction (31, 62). The p-value determined by 2-tailed Student t-test comparing with and without MDM2 knockdown was p-value=0.01. Two independent scorers counted the numbers of masses for each independent experiment. **(B)** A representative image from confocal immunofluorescence microscopy showing a single slice from z-stack of DAPI, GFP and F-Actin of estrogen treated inducible clonal T47D.sh*mdm2* cells grown in 3D-matrigel in the presence or absence of 4μg/ml doxycycline (dox) for 3 weeks. The top and middle rows show hollow lumen and ductal lumen respectively in the presence of shRNA expression to *mdm2*; the GFP (green) indicates shRNA induction to *mdm2*. The third row shows mass structure (disruption of normal mammary glandular architecture) in the absence of shRNA expression to *mdm2*.

**Figure 3:**
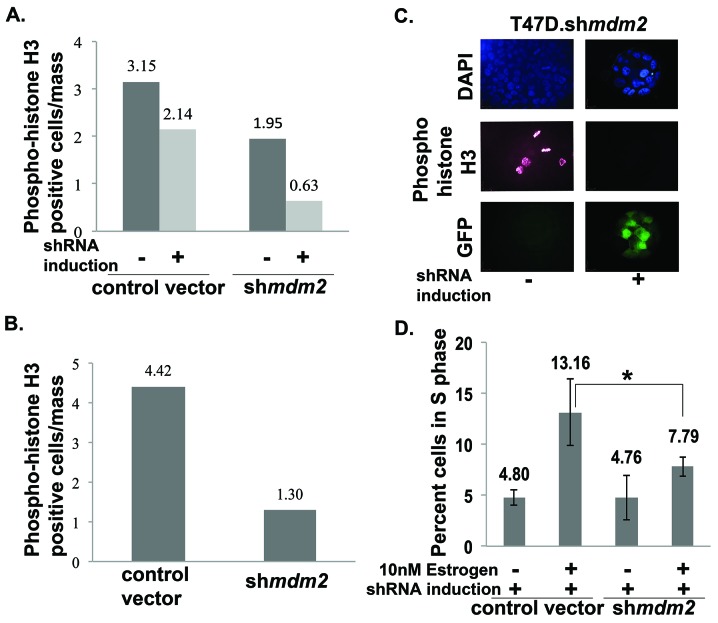
MDM2 knockdown impedes mitosis and significantly decreases the number of estrogen-driven S phase cells **(A)** & **(B)** T47D cells ((A) inducible sh*mdm2* clonal and vector pool; (B) constitutive sh*mdm2* and vector pool) grown in the presence of estrogen in 3D matrigel for 3.5 weeks. The cells were fixed, permeabilized, blocked, stained with phospho-histoneH3 antibody and mounted with DAPI containing mounting media. Images were taken with confocal microscope. Quantitative analysis of phospho-histone H3 positive cells were performed by capturing optical Z-stack sections of masses and dividing the number of positive phospho-histone H3 cells by the total number of masses. (A) The number of masses counted in each group for 2 independent experiments were control vector -shRNA induction (29, 30), control vector +shRNA induction (30, 30), *mdm2*shRNA -shRNA induction (29, 30) and *mdm2*shRNA +shRNA induction (30, 30). (B) The number of masses counted in each group for 2 independent experiments were control vector (49, 60) and sh*mdm2* (48, 60). Average of two independent experiments are shown for each knockdown method. The p-value determined by 2-tailed Student t-test comparing with and without MDM2 knockdown was p-value=0.001 (A) and p-value=0.009 (B). Two independent scorers counted the numbers of masses for each independent experiment. **(C)** Representative confocal Z-stack image (single slice) showing DAPI, phospho-histone H3 and GFP in estrogen-treated inducible clonal T47D.sh*mdm2* cells in the presence and absence of 4μg/ml doxycycline. **(D)** Cell cycle analysis by FACS (Fluorescence Activated Cell Sorting). T47D cells were harvested, fixed and stained with propidium iodide and subjected to cell cycle analysis by FACS. Data are presented as percent of cells in S phase in a total population of 10,000 cells and analyzed by FACS in each group. Average of 4 independent experiments are shown. The p-value was determined by 2-tailed Student t-test and * represents a p-value ≤ 0.05

**Figure 4:**
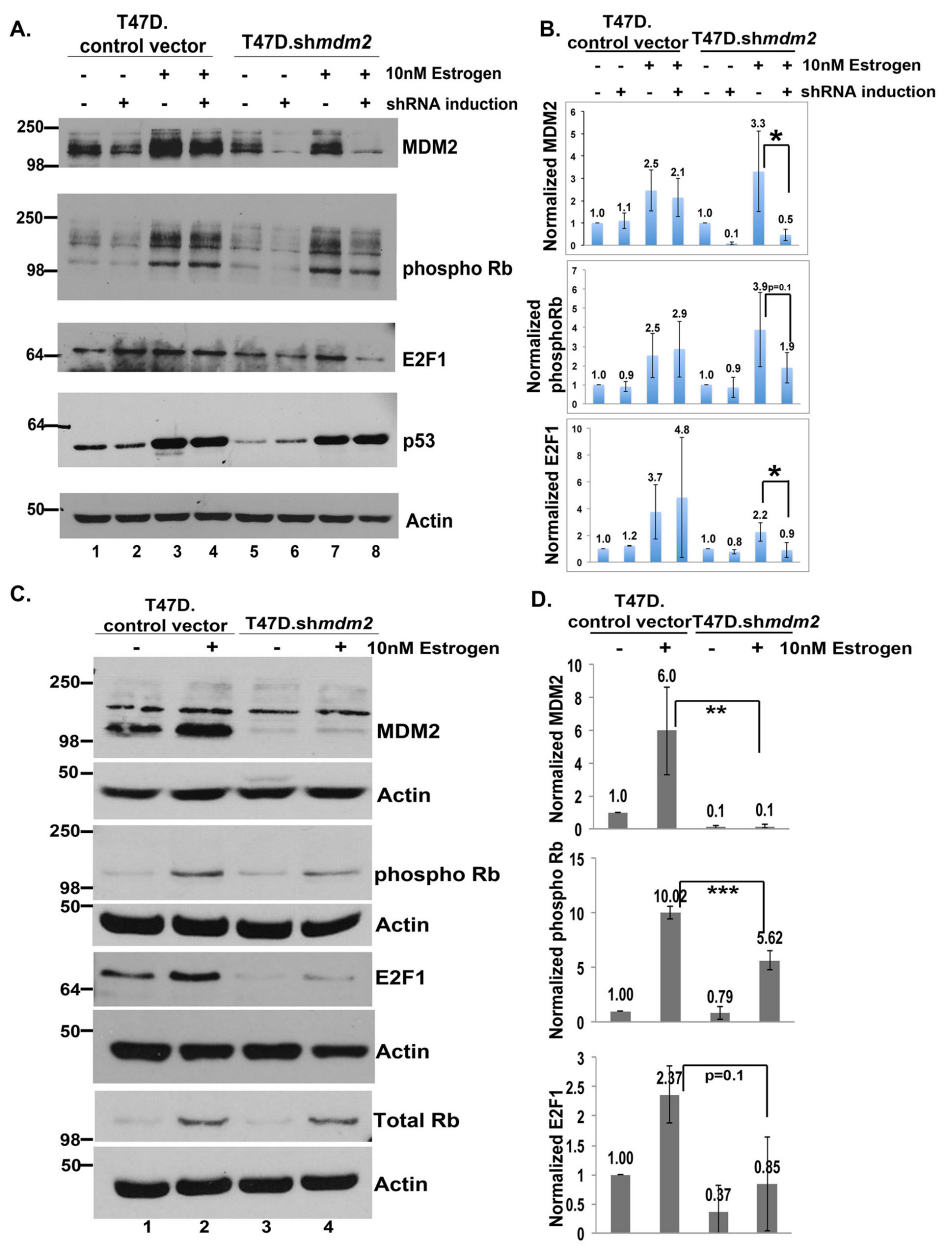
In ER+ T47D breast cancer cells MDM2 influences estrogen mediated cell proliferation through the Rb-E2F1 pathway **(A)** Inducible clonal T47D cells with *mdm2* shRNA or control vector were treated with or without 4μg/ml doxycycline (dox) for 3 days, followed by 10nM estrogen for 5 days in the presence or absence of dox. A representative image of western blot analysis of MDM2, phospho Rb, E2F1, p53 and Actin protein levels from 50μg whole cell protein extract is shown. **(B)** ImageJ analysis was performed for MDM2, phospho Rb and E2F1 protein levels normalized to Actin. The graph represents an average of three independent experiments with standard deviation in inducible clonal T47D cells with *mdm2* shRNA or control vector. **(C)** A constitutive pool of T47D cells with *mdm2* shRNA or control vector were grown with or without 10nM estrogen for 5 days. A representative image of western blot analysis of MDM2, phospho Rb, E2F1, total Rb and Actin protein levels from 50μg whole cell protein extract is shown. **(D)** ImageJ analysis was performed for MDM2, phospho Rb and E2F1 protein levels normalized to Actin. Graph represents average of three independent experiments with standard deviation in constitutive pool of T47D cells with *mdm2* shRNA or control vector. * represents a p-value ≤ 0.05, ** represents a p-value ≤ 0.01, *** represents a p-value ≤ 0.001. The p-value was determined by 2-tailed Student t-test.

**Figure 5:**
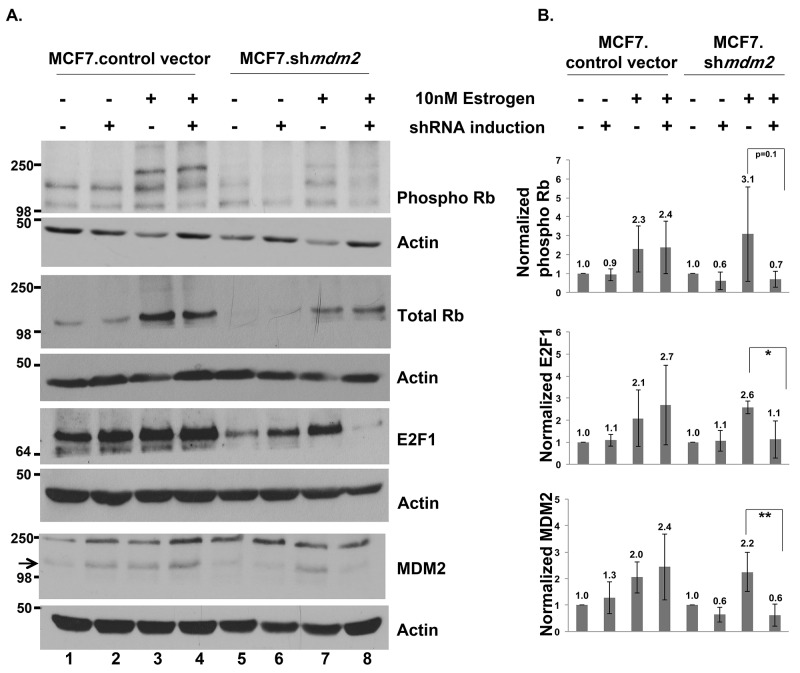
In ER+ MCF7 breast cancer cells MDM2 influences estrogen mediated cell proliferation through the Rb-E2F1 pathway **(A)** Inducible clonal MCF7 cells with *mdm2* shRNA or control vector were treated with and without 2μg/ml doxycycline(dox) for 3 days to induce shRNA expression, followed by 10nM estrogen for 5 days in the presence and absence of dox. A representative image of western blot analysis of phospho Rb, Total Rb, E2F1, MDM2 and Actin protein levels from 50μg whole cell protein extract is shown. **(B)** ImageJ analysis was performed for phospho Rb, E2F1 and MDM2 protein levels normalized to Actin. Graph represents average of four independent experiments with standard deviation in inducible clonal of MCF7 cells with *mdm2* shRNA or control vector. * represents a p-value ≤ 0.05, ** represents a p-value ≤ 0.01. The p-value was determined by 2-tailed Student t-test.

**Figure 6:**
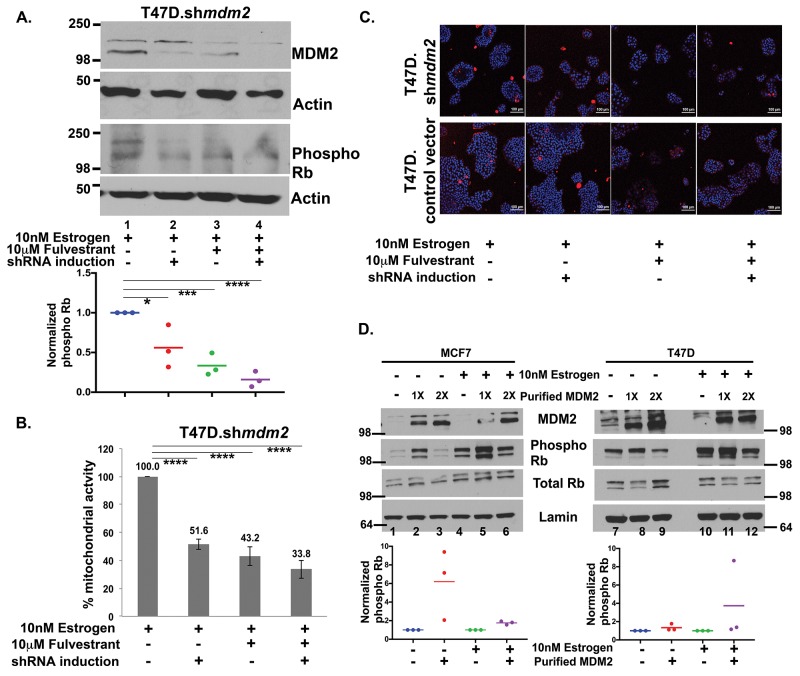
Fulvestrant treatment inhibits MDM2 expression and blocks the Rb-E2F1 pathway **(A)** T47D.sh*mdm2* cells were treated with 10nM estrogen (lane 1) and either had MDM2 knockdown (lane 2) or 10μM fulvestrant treatment (lane 3), or both (lane 4) for 5 days. A representative western blot analysis of MDM2, phosphoRb and Actin protein levels from 50μg whole cell protein extract is shown. Dot plot diagram shows quantified ImageJ values of phospho Rb protein levels normalized to Actin from three independent experiments. **(B)** MTT assay was performed in T47D.control vector and inducible T47D.sh*mdm2* clonal cell lines after treatments. Percentage mitochondrial activity represents an average of 2 independent experiments. (A) & (B) * represents a p-value ≤ 0.05, *** represents a p-value ≤ 0.001, **** represents a p-value ≤ 0.0001. The p-value was determined by 2-tailed Student t-test. **(C)** A representative live cell image by confocal microscopy with 20X objective of T47D.vector and inducible clonal T47D.sh*mdm2* clonal cell lines after treatments. Red fluorescence represents staining with propidium iodide. Blue fluorescence represents staining of nuclear DNA. **(D)** MDM2 drives phosphorylation of Rb in ER+ breast cancer cells. *In vitro* kinase assay was performed to detect phosphorylation of Rb with or without overnight estrogen treatment in either presence or absence of bacterially expressed and purified MDM2 (1μl or 2μl). A representative image of Western blot analysis of MDM2, phospho Rb and total Rb protein level from nuclear extract of MCF7 (left) and T47D (right) cells are shown. Dot plot diagram shows quantified ImageJ values of phospho Rb protein levels normalized to lamin A from three independent experiments, when 1μl of purified MDM2 was added to the nuclear extracts of MCF7 (left) and T47D (right) cells. The p-value for MCF7 cells with overnight estrogen treatment and addition of purified MDM2 (compare lane 4 to lane 5) was statistically significant. The p-value was determined by 2-tailed Student t-test.

Matrigel conditions allow cells to recapitulate *in vivo* mammary glandular architecture [30]. We previously documented that MDM2 knockdown in MCF7 and T47D cells leads to decreased proliferation and for MCF7 cells a reduction in large size masses in matrigel [11]. We studied the p53-independent role of MDM2 on T47D colonies formed in 3D matrigel culture conditions to determine the biological significance of estrogen signaling through the MDM2 pathway. Reduced MDM2 caused a decrease in matrigel colony size (Figure 1D), and this agreed with the results presented for reduction of large soft agar colony formation shown in Figure 1A and 1B. We stratified the T47D masses into 5 different categories (Figure 1D, very small (yellow), small (red), intermediate (green), large (purple) and very large (blue)). MDM2 knockdown caused a robust decrease in large and very large masses and an increase in very small and small masses. The T47D.vector control cell line did not produce small masses. The clonal T47D.sh*mdm2* cell line had some very small and small masses even without shRNA induction. This leaky phenotype of the T47D.sh*mdm2* was reproducible, and documented by two independent scorers for two independent experiments. We saw MDM2 knockdown decreased colony size of MCF7 and T47D cells in two mechanistically different experiments, repeated multiple times, and with multiple scored colonies.

### MDM2 knockdown in T47D cells grown in matrigel promotes lumen formation

When T47D cells are grown in matrigel, they have a cancer mass morphology with disrupted architecture and filled lumen [30]. From the average of two independent experiments we observed that MDM2 depletion reverted this phenotype to become more normal (Figure 2). With *mdm2* knockdown, and not in vector induced control samples, we observed a significant average increase in the number of masses having hollow ductal lumen architecture that showed a degree of luminal clearance (Figure 2A). We observed different luminal clearance phenotypes. A small proportion had perfect hollow lumen architecture (representative image shown in Figure 2B, top row), and many had variations with multiple hollow regions (representative image shown in Figure 2B, middle row). When MDM2 was not depleted there were significantly less masses with hollow lumen (Figure 2A see vector control results and MCF7.*shmdm2* no induction results and Figure 2B, bottom row for representative image). This result was recapitulated in the ER+ MCF7 breast cancer cells (Supplementary Figure 1A and 1B). This shows that in some ER+ breast cancer cells the knockdown of MDM2 can revert the tumorigenic 3D filled lumen phenotype to a hollow lumen phenotype. The fact that this occurs in T47D cells with mtp53 supports our earlier finding that the estrogen-MDM2 axis works, at least in part, through a wild-type p53-independent pathway [11].

### MDM2 knockdown inhibits mitosis in 3D cell culture condition

Mitosis-specific phosphorylation of histone H3 can be used as an indicator of cells undergoing mitosis [31]. We asked if the luminal clearance phenotype associated with decreased mitotic cells in matrigel masses. To do this we carried out two biological replicates for two different knockdown strategies and probed the masses with mitosis-specific anti-phospho-histone H3 followed by analysis with confocal microscopy. MDM2 knockdown resulted in a dramatic decrease in the average number of mitotic cells observed in matrigel masses derived from T47D cells with inducible MDM2 knockdown (Figure 3A) and masses derived from T47D cells with constitutive MDM2 knockdown (Figure 3B). A representative image of inducible clonal T47D.sh*mdm2* cells is shown in Figure 3C. In MCF7 cells with MDM2 knockdown we also observed a similar decrease in cells staining with anti-phospho-histone H3 (Supplementary Figure 1C and 1D). This supports several studies that have shown MDM2 overexpression drives cell cycle progression into S phase of the cell cycle [18, 32]. We tested the hypothesis that the estrogen-MDM2 axis drives the T47D cells into S phase by carrying out fluorescence activated cell sorting (FACS) on cells grown in standard 2D culture with estrogen with and without MDM2 depletion (Figure 3D). As predicted, estrogen treatment increased the number of S phase cells detected by FACS analysis and MDM2 knockdown significantly reduced the S phase percentage (Figure 3D). Together our data suggest MDM2 up-regulation is critical for the estrogen-driven S-phase entrance as well as the disruption of mammary architecture. Interestingly, from four independent biological replicates with MDM2 knockdown the S phase was the only stage that showed a statistically significant reduction.

### The estrogen-mediated proliferative advantage is through an MDM2-Rb-E2F1 pathway

E2F1 is known to promote cell cycle progression so we tested if Rb phosphorylation and E2F1 protein levels were changed by estrogen treatment and if MDM2 knockdown caused reversion. Estrogen treatment of T47D cells reproducibly increased MDM2 protein and this was reduced by sh*mdm2* induction to knockdown MDM2 (Figure 4A, lanes 1-8 top panel). Importantly estrogen treatment increased phosphorylated Ser807/Ser811 Rb protein levels (which correlates with activation of the E2F pathway) and this phosphorylation was decreased by MDM2 knockdown (Figure 4A, lanes 1-8 phospho Rb panel). We detected a reduction of the lower 110 kD phospho Rb band as well as multiple higher migrating forms (Figure 4A, phospho Rb panel, compare lanes 7 and 8). The MDM2 knockdown also resulted in a decrease in E2F1 protein (Figure 4A, E2F1 panel, compare lanes 5 and 6, and lanes 7 to 8). Estrogen treatment caused an increase in the level of mtp53 and this mtp53 level was unchanged by the knockdown of MDM2. We used imageJ analysis to quantify the levels of MDM2, phosphorylated Ser807/Ser811 Rb, and E2F1 from three independent biological replicates for T47D.sh*mdm2* western blots (Figure 4B). MDM2 knockdown caused a statistically significant reduction of MDM2 and E2F1 and a strong reduction in phosphorylated Rb that was not statistically significant. Using a different knockdown method we further validated the estrogen-MDM2-Rb-E2F1 axis from three biological replicates of the T47D cells with constitutive MDM2 knockdown (Figure 4C and 4D). For this alternative knockdown method we observed that constitutive MDM2 knockdown resulted in a statistically significant decrease in the estrogen-driven phosphorylation of Rb. In these conditions we detected only the predominant lower Rb band at 110 kD (Figure 4C). This detection of only the lower Rb form was due to a lower exposure of the western blot.

We investigated the estrogen-MDM2 axis signaling to Rb in MCF7 cells. We previously published that exposing MCF7.*shmdm2* cells to estrogen increases the amount of MDM2 protein level and depletion of the MDM2 does not increase the p53 protein level [11]. In this experiment, in support of the T47D data, estrogen treatment of the control and MCF7.*shmdm2* cells increased MDM2, phosphorylated Rb, total Rb and E2F1 (Figure 5A and 5B) and induction of *mdm2* shRNA reduced MDM2 protein, phosphorylated Rb and E2F1 protein levels (Figure 5A compare lanes 3 and 4 to lanes 7 and 8 and see 5B). ImageJ analysis of four independent biological replicates with MDM2 knockdown indicated a statistically significant reduction in E2F1 and a strong reduction in phosphorylated Rb (Figure 5B).

### Fulvestrant decreases MDM2 levels and blocks phosphorylation of Rb

Fulvestrant is a clinically used ER antagonist that promotes MDM2 protein turnover [33]. MDM2 has been shown to promote tamoxifen resistance and therefore combination-fulvestrant treatments have been proposed for patients with advanced breast cancer as a way to target the MDM2 axis [13, 33]. We investigated if fulvestrant could block the estrogen-MDM2-mediated phosphorylation of Rb. We observed that fulvestrant treatment of T47D.*shmdm2* cells downregulated both MDM2 and phosphorylated Rb levels (Figure 6A, compare lanes 1 and 3). This down regulation was slightly improved by sh*mdm2* induction (Figure 6A, phospho Rb panel, lane 4). This decrease was observed in three independent biological replicates with fulvestrant alone showing a statistically significant decrease and the combination of MDM2 knockdown plus fulvestrant showing a trend for further reduction (Figure 6A, dot plot of the three experiments shown below western blot). We were interested in examining whether the combination of fulvestrant and MDM2 knockdown also caused a further reduction of cell viability compared to fulvestrant treatment alone. We saw that in the presence of estrogen the mitochondrial activity was statistically significantly reduced by either MDM2 knockdown or fulvestrant treatment almost to the same extent (Figure 6B). When cells were subjected to MDM2 knockdown and fulvestrant treatment together there was more decrease in mitochondrial activity than with either alone, but this was not statistically significant (Figure 6B). Therefore fulvestrant effectively blocks the estrogen-MDM2-mediated axis. We wondered if estrogen signaling to MDM2 increased proliferation by blocking cell death. We carried out live cell imaging to determine if either MDM2 knockdown, or fulvestrant treatment, increased cell death (Figure 6C, blue staining detects nuclei and red staining showing dead cells). Neither MDM2 knockdown nor fulvestrant treatment resulted in an increase in dead cells further supporting that the pathway targeted by the estrogen-MDM2 axis was involved in cell cycle regulation (Figure 6C T47D.*shmdm2* and T47D vector control).

### MDM2 drives Rb phosphorylation

Our data suggested that the up-regulation of MDM2 might have a direct role in increasing the phosphorylation of Rb because estrogen stimulation of MCF7 and T47D cells required MDM2 for the increased phosphorylation of Rb. We wondered if MDM2 protein directly promoted signal transduction for increased phosphorylation of Rb. To test this we performed an *in vitro* kinase assay with the addition of bacterially purified MDM2 to MCF7 and T47D cancer cell extract from cells grown with or without overnight estrogen treatment. We observed that MCF7 cell extract (Figure 6D on the left) supported increased phosphorylation of Rb in three independent biological replicates with a lower but not higher amount of MDM2 (Figure 6D, left side and dot plot for low MDM2 addition shown below). The increased MDM2 did not decrease total Rb so less phosphorylation of Rb might be due to other alterations in post-translational modifications but not degradation. We observed a similar (but less robust) result in T47D extract (Figure 6D, right side). Taken together, our data suggest that estrogen provokes signals to increase MDM2 expression and this estrogen stimulated MDM2 promotes signal transduction for increasing phosphorylation of Rb.

## DISCUSSION

The p53-independent oncogenic functions of MDM2 are less well understood than the MDM2 canonical wild-type p53 feedback pathway. Many breast cancers contain no functional wild-type p53 and have high expression of MDM2. MDM2 overexpression is directly related to ER alpha overexpression [2, 4, 13, 34]. A recent analysis of The Cancer Genome Atlas (TCGA) invasive breast cancer data found significantly higher *mdm2* mRNA in ER alpha-positive tumors without a correlation to p53 wild-type or mutant status [13]. This analysis also showed that a high ER alpha level correlates with increased expression of both MDM2 and MDM4 and that ER alpha-targeting therapies down-modulate both MDM2 and MDM4 expression [13]. We, and others, have observed that estrogen provokes p53-independent proliferative effects [10, 11, 13, 33]. In this paper we present data that suggests that the estrogen-MDM2 axis signals a cascade resulting in the phosphorylation of Rb. Massive amounts of publicly available breast cancer data are available to evaluate the *in vivo* breast cancer relationships between MDM2 and phosphorylated Rb [35]. We used the cBioportal http://www.cbioportal.org/ with the TCGA breast cancer data from http://cancergenome.nih.gov/ to evaluate the correlation between MDM2 protein expression and phosphorylated Rb in patient tumor tissue (Supplementary Figure 2). We observed a positive correlation between phosphorylated Rb and MDM2 protein expression (Pearson r = 0.192; p-value=0.0638) and a negative correlation between total Rb and MDM2 protein expression (Pearson r = -0.108; p-value=0.2983). The correlation between MDM2 and phosphorylated Rb was strengthened by the fact that high MDM2 did not have a strong correlation with changes in total Rb. This argues for a true relationship between increased MDM2 in breast cancers and the activation of the Rb-E2F1 pathway. Our data support that an estrogen-MDM2 axis activates the Rb-E2F1 pathway in at least a subset of breast cancers.

**Figure 7:**
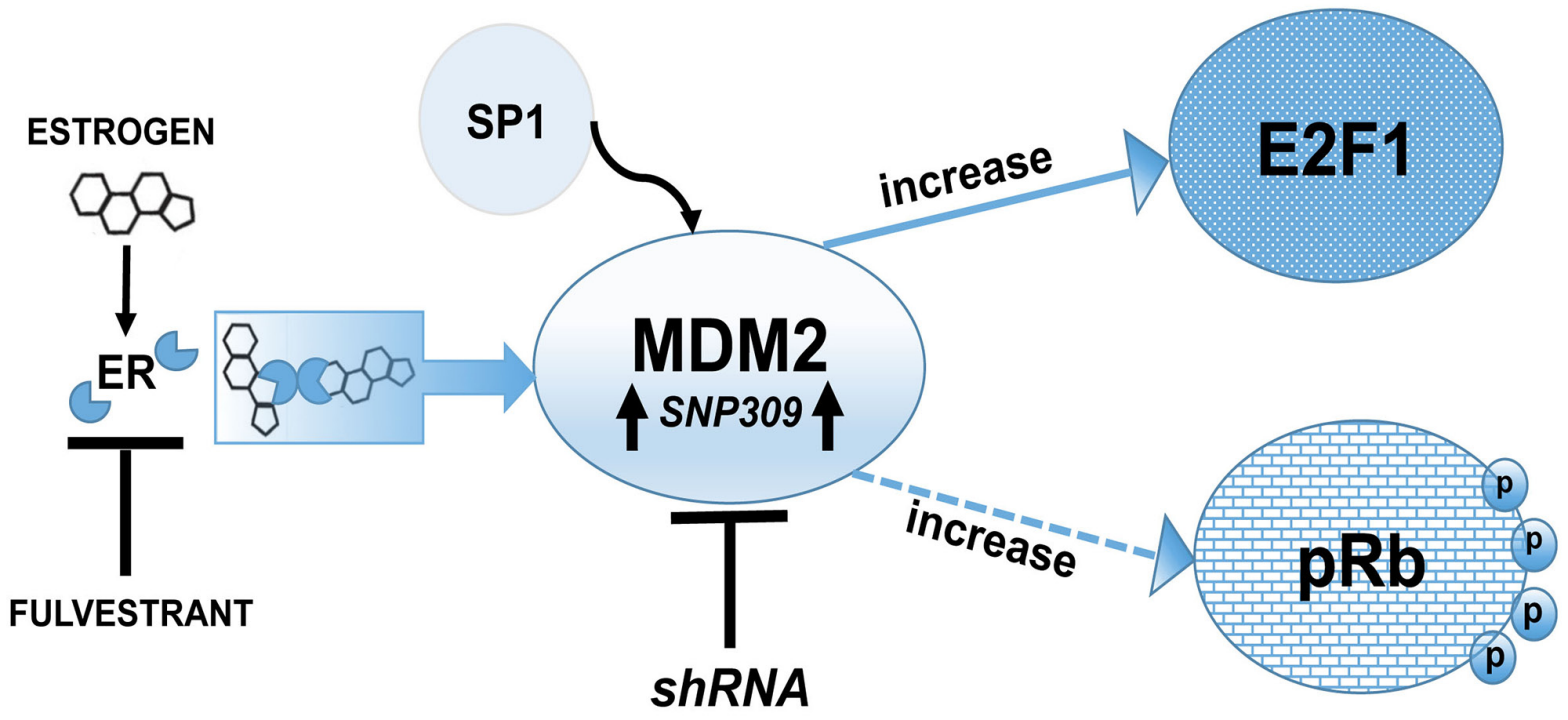
Schematic representation of MDM2 signaling pathway showing a p53-independent function of MDM2 is required for estrogen mediated breast cancer proliferation and disruption in 3D mammary architecture Model showing that MDM2 is a central hub in estrogen signaling and works through an Rb-E2F1 pathway to promote proliferation. Breast cancer cells harboring SNP309 have increased binding of the transcription factor Sp1, which causes elevated MDM2 protein levels. Fulvestrant blocks the MDM2 pathway and the Rb-E2F1 pathway.

We examined estrogen-driven breast cancer cell growth in 3D culture and asked if MDM2 was necessary for the hormone-dependent growth properties. Interestingly we found that growth of MCF7 and T47D cells in soft agar in the presence of estrogen required MDM2 expression. The knockdown of MDM2 prevented cells with either wild-type p53 or mutant p53 from achieving estrogen-mediated anchorage independent cell growth. This not only demonstrated that estrogen was signaling through up-regulation of MDM2 for mammary tumorigenic related phenotypes but also that MDM2 provided this function in the absence of promoting the degradation of p53. We studied the p53-independent MDM2-mediated cell morphology in more depth by examining the influence of MDM2 on estrogen-mediated growth in a laminin rich matrigel environment. We observed that MDM2 expression was required for the lack of luminal clearance that associates with breast cancer cell growth in matrigel and that knockdown of MDM2 resulted in luminal clearance. This is the first report of estrogen working through an MDM2 pathway, which leads to the disruption of mammary architecture, and signaling for the phosphorylation of Rb (see model in Figure 7). Our preliminary data with the triple negative breast cancer (TNBC) cell line MDA-MB-231 suggests no influence of MDM2 on proliferation in the absence of receptor signaling. The knockdown of MDM2 in estrogen receptor negative (ER-) MDA-MB-231 cells did not decrease cell proliferation (Supplementary Figure 3). Therefore in T47D and MCF7 cells there may be some MDM2-mediated cross-talk with estrogen driven factors.

Estrogen signaling has been shown to up-regulate Rb phosphorylation [25] and MDM2 decreases total Rb while increasing phosphorylated Rb [21, 26]. However there has never been a connection made showing that the estrogen axis requires signaling to MDM2 for Rb phosphorylation thus indicating an estrogen-MDM2-Rb-E2F1 axis. We observed that MDM2 was required in T47D and MCF7 cells in order for estrogen to stimulate the phosphorylation of Rb. Interestingly while we observed an increase in phosphorylated Rb, we did not observe a decrease in total Rb. We have demonstrated for the first time that there is an estrogen-MDM2-Rb-E2F1 axis in some breast cancer cells.

Fulvestrant is a novel hormone receptor antagonist that causes rapid degradation of both the ER and the progesterone receptor (PgR) [36] and has been shown to be highly effective for treating late stage breast cancer [37]. Mechanistically, fulvestrant can block the estrogen mediated increased in MDM2 [13, 33]. We observed that in addition to blocking the up-regulation of MDM2, fulvestrant also downregulated Rb phosphorylation. Moreover, the combination of fulvestrant with MDM2 knockdown caused a slight reduction in phosphorylated Rb beyond fulvestrant alone (although not statistically significant). We observed that the addition of MDM2 to breast cancer cell extracts *in vitro* modestly increased the phosphorylation of Rb. However, only low and not high MDM2 increased Rb phosphorylation. It is possible that a fine tuned regulation of ubiquitination activates Rb phosphorylation while increased ubiquitination blocks Rb phosphorylation. MDM2 has been shown to ubiquitinate Rb and the level of ubiquitination is reduced by the deubiquitinase HAUSP [38]. MDM2 knockdown and fulvestrant treatment reduced Rb phosphorylation. Fulvestrant blocks estrogen-mediated increases in MDM2 by increasing MDM2 protein turnover [33]. Fulvestrant alone causes G1 cell cycle arrest that is p53-independent and our live cell confocal microscopy indicated that this fulvestrant and/or MDM2 knockdown did not increase cell death. Our data supports the use of fulvestrant as a way to target the estrogen-MDM2 signal transduction pathway. Combination of fulvestrant with doxorubicin, etoposide or paclitaxel is synergistic for inhibiting breast cancer proliferation and induces cell death by suppressing MDM2 expression [33]. The results presented here support the idea that targeting the MDM2 pathway by fulvestrant treatment of estrogen-receptor positive breast cancers that are not resistant to fulvestrant may be an excellent mechanism to target the cancer at multiple estrogenic hubs. New MDM2 targeting agents are in clinical trials. Fulvestrant resistant ER-positive breast cancers have reduced ER and increased ERBB2 [39]. Such resistant breast cancers pose a challenge and it will be interesting to see if fulvestrant resistant breast cancers have up-regulation of MDM2. If fulvestrant-resistant cancers have up-regulated MDM2 they might be excellent candidates for treatment with MDM2 blocking therapies.

## MATERIALS AND METHODS

### Cell culture

2D culture: Human breast cancer cells T47D (*mdm2* SNP309 G/G, mutant p53 L194F), MCF7 (*mdm2* SNP309 T/G, wild-type p53), and MDA-MB-231 (mdm2 SNP309 T/G, mutant p53 R280K) cells were obtained from American Type Culture Collection (ATCC). They were grown in DMEM (Invitrogen), supplemented with 10% fetal bovine serum (FBS, Gemini) and 50U/ml penicillin and 50μg/ml streptomycin (Mediatech) at 5% CO_2_ in a 37°C humidified incubator. Cell lines with the inducible *mdm2* knockdown were generated as described previously [11]. A T47D cell pool with constitutive knockdown of *mdm2* was generated for this work by infection with MLP vector (a generous gift from Scott Lowe) containing *mdm2* 151656 shRNA or empty vector by the retroviral mediated gene transfer method. In short, empty or sh*mdm2* containing vector were transfected using the calcium phosphate method to Phoenix packaging cells to make virus that was used to infect T47D cells and then they were selected with 5μg/ml puromycin.

### Anchorage independent growth assay

Cells were grown in 2D culture for 3 days with or without doxycycline. They were trypsinized, washed with 1X clear HBSS three times and then counted. Cells with equal seeding densities were mixed with 0.3% Noble Agar (Sigma A5431) in phenol-red-free-DMEM media containing 10% charcoal stripped FBS (Gemini), antibiotics, 10nM estrogen and with or without doxycycline. They were seeded onto 35mm dishes coated with 0.5% Nobel agar in the same media. They were then toped with the same media. Estrogen-treated growth medium with or without doxycycline was replaced every 3 days. Colonies were counted after 3-3.5 weeks.

### Cell culture in matrigel

Cells were grown in 2D culture for 3 days with or without doxycycline. They were trypsinized, washed with 1X clear HBSS three times and then counted. They were seeded on top of 40μl solidified matrigel (Cultrex) in phenol-red-free-DMEM media containing 10% charcoal stripped FBS (Gemini), antibiotics, 10nM estrogen and with or without doxycycline. Medium was changed every 3 days. Masses were grown for 3-3.5 weeks, fixed with 4% paraformaldehyde, permeabilized, blocked and stained with propidium iodide or actin immunostaining by Rhodamine-Phalloidin (Cytoskeleton BK005) or primary antibody phospho histone H3 (Millipore) followed by Alexa Fluor conjugated secondary antibody (Invitrogen) and mounted with Vectashield mounting medium containing DAPI (Fisher Scientific). Imaging was performed using confocal microscope using laser scanning spectral confocal microscope TCS SP2.

### Treatments

Inducible sh*mdm2* or vector control cell lines were grown in complete media described above, with or without doxycycline (4μg/ml for T47D, and 2μg/ml for MCF7, and 6μg/ml for MDA-MB-231) for shRNA induction for 3 days. The growth medium was then replaced with phenol-red-free DMEM media containing 10% charcoal stripped FBS (Gemini) and antibiotics and treated with 10nM estrogen (17-beta-estradiol, Sigma) and/or 10μM fulvestrant (Sigma-Aldrich) wherever applicable. Fresh medium was applied every 72 hours.

### MTT assay

Cell viability was often measured as mitochondrial activity using tetrazolium dye-based microtitration assay. Cells were seeded at 20 × 10^3^ per well in 12-well plate. After treatment, MTT (3-(4,5-Dimethylthiazol-2-yl)-2,5-diphenyltetrazolium bromide) assay was performed as per manufacturers instructions (Sigma). The absorbance was quantified by measuring absorbance at 550nm (the 620nm absorbance was subtracted from the background). Data represent percentage mitochondrial activity in each sample relative to the untreated sample.

### Live cell imaging

Cells were seeded at 20 × 10^3^ per well in a 12-well glass bottom plate (MatTek, Ashland, MA, USA). After treatment, cells were stained with 50 ul ReadyProbes Cell Viability Imaging Kit Blue/Red (Life Technologies Cat# R37610) for 15 min at room temperature and z-stack images of stained cells were taken by confocal microscopy using a Nikon A1 confocal microscope with 20x objective. Maximum projection images are shown. Propidium iodide: red fluorescence; Nuclear DNA: blue fluorescence.

### Fluorescence activated cell sorting (FACS)

FACS was performed on FACScan device (BD Biosciences). After treatments, cells were harvested, washed, resuspended in PBS containing 2% bovine serum albumin, 0.1% sodium azide, fixed in 30% ethanol and stored overnight at 4°C. Before sorting, propidium iodide staining and RNAse treatment were performed for 30 minutes at 37°C.

### Whole cell protein extract

Cells were pelleted from 2D culture at 1100 rpm for 7 mins at 4°C and washed 3 times with 1X ice-cold PBS. The cells were then suspended in RIPA buffer (0.1% SDS, 1% IGEPAL NP-40, 0.5% Deoxycholate, 150mM NaCl, 1mM EDTA, 0.5mM EGTA, 50mM Tris-Cl pH8) with 1mM PMSF, 8.5μg/ml Aprotinin, 2μg/ml Leupeptin, sodium fluoride (5mM), sodium orthovanadate (1mM) and phosphatase cocktail inhibitor (Sigma) following standard protocol.

### Purification of MDM2 expressed in bacteria

After IPTG induction for 4 hours, BL21DE3 (having plasmid pRSETA HDM2; generous gift from Lindsey Mayo lab) bacterial cell pellets were collected and lysed with lysis buffer (pH 8: 100mM Na_2_H_2_PO_4_, 10mM Tris-Cl, 8M Urea, 1mM PMSF) by frequent vortexing. It was then centrifuged for 25 minutes in 4°C at 10,000g. The supernatant was collected, mixed with Ni-NTA agarose beads (Qiagen, #30210) in the ratio of 1:3 (bead to supernatant) and rocked for an hour at 4°C. After a quick spin as the beads settled, the supernatant was transferred and labeled as “unbound”. The pellet was washed with wash buffer (pH 6.3: 100mM Na_2_H_2_PO_4_, 10mM Tris-Cl, 8M Urea, 1mM PMSF) in 1:2 (bead to buffer) 2 times and the supernatant was labeled as “wash”. The remaining pellet was mixed in 2.5 X the elution buffer (pH 5.9: 100mM Na_2_H_2_PO_4_, 10mM Tris-Cl, 8M Urea, 100mM EDTA pH8, 1mM PMSF) and supernatant was collected after quick spun and labeled as “elute”. This was repeated 2 more times. Small aliquots were taken at each elution and run on a gel to confirm purification of MDM2 compared to wash and unbound. The third elute of purified MDM2 was used in *in vitro* kinase assay.

### Nuclear extracts

Pellets were collected of cells with and without overnight estrogen treatment. The pellets were suspended in 5X packed cell volumes with cytoplasmic extraction buffer (CEB: 10mM Hepes pH 7.9; 1.5mM MgCl_2_; 10mM KCl; 0.5mM PMSF, 0.5 mM DTT; 8.5μg/ml Aprotinin, 2μg/ml Leupeptin; and phosphatase inhibitor mixture I (Sigma)). After washing, cells were resuspended with a 20-gauge needle in 2X packed cell volumes of CEB and incubated on ice for 10 min. After centrifugation (10 min at 12,000 rpm at 4 °C) the supernatant was removed to give the cytoplasmic fraction. The pellet was resuspended in kinase reaction buffer (20 mmol/L Tris pH 7.5; 7.5 mmol/L MgCl_2_; 1 mmol/L DTT; 0.5 mmol/L ethyleneglycol-bis [beta-aminoethyl]-*N*,*N*,*N*′,*N*′-tetraacetic acid [EGTA]; 25 mmol/L beta-glycerophosphate; 0.5 mmol/L sodium orthovanadate; 1 mmol/L PMS; 2 μg/ml leupeptin, 1.5 μg/ml aprotinin and phosphatase inhibitor mixture). Cells were resuspended with a 20-gauge needle and the cell suspension was rocked for 30 min at 4 °C and then centrifuged for 30 min at 13,000 rpm at 4 °C. The supernatant was the nuclear fraction.

### *In vitro* kinase assay

The breast cancer cell nuclear extract made in kinase buffer was incubated with 1μl or 2μl of purified MDM2 in elution buffer or elution buffer alone (control) and ATP regeneration system (1mM ATP, 1mM magnesium chloride, 10mM phosphocreatine, 50μg/ml creatine kinase) for 30 minutes at 30°C. The total reaction volume was 50μl. The reaction was terminated with 4X NuPAGE Lithium dodecyl Sulfate buffer (Life Technologies) and western blot assay was performed immediately.

### Western blot assay

Protein extracts from 2D culture were prepared with 4X NuPAGE Lithium dodecyl Sulfate buffer (Life Technologies) and 20mM DTT. The samples were heated at 70°C for 10 min and then 100 mM Iodoacetamide (Sigma) was added after heating. Samples were separated by SDS-PAGE followed by electrotransfer onto a nitrocellulose membrane. The resulting membrane was blocked with 5% non-fat milk (Biorad) in 1X PBS-0.1% Tween-20 or 5% Bovine Serum Albumin (BSA) in 1X TBS-0.1% Tween-20 (for probing phospho Rb) and probed with primary antibody overnight at 4°C. The membrane was washed with 1X PBS-0.1% Tween-20 or 1X TBS-0.1% Tween-20 followed by probing with secondary antibody. The protein signal was detected by chemiluminescence using the Super Signal Kit (Pierce) and autoradiography using Hyblot CL films (Denville Scientific).

### Antibodies

AntiMDM2 antibody – 1:1:1 mix of mouse monoclonal antibodies 4B2, 2A9, 4B11; antip53 antibody - 1:1:1 mix of mouse monoclonal antibodies 240,421,1801; rabbit polyclonal antiActin (Sigma-Aldrich) or anti-Actin HRP (Santa Cruz); phospho Rb (Ser807/811: Cell Signaling); Total Rb (Cell Signaling); E2F1 (Cell Signaling) and secondary anti-mouse and anti-rabbit antibodies (Sigma-Aldrich) were used for western blot analysis.

## SUPPLEMENTARY MATERIALS AND FIGURES



## References

[1] Jordan VC, O’Malley BW (2007). Selective estrogen-receptor modulators and antihormonal resistance in breast cancer. J Clin Oncol.

[2] Hori M, Shimazaki J, Inagawa S, Itabashi M (2002). Overexpression of MDM2 oncoprotein correlates with possession of estrogen receptor alpha and lack of MDM2 mRNA splice variants in human breast cancer. Breast Cancer Res Treat.

[3] Cancer Genome Atlas Network (2012). Comprehensive molecular portraits of human breast tumours. Nature.

[4] Marchetti A, Buttitta F, Girlando S, Dalla Palma P, Pellegrini S, Fina P, Doglioni C, Bevilacqua G, Barbareschi M (1995). mdm2 gene alterations and mdm2 protein expression in breast carcinomas. J Pathol.

[5] Onel K, Cordon-Cardo C (2004). MDM2 and prognosis. Mol Cancer Res.

[6] Rayburn E, Zhang R, He J, Wang H (2005). MDM2 and human malignancies: expression, clinical pathology, prognostic markers, and implications for chemotherapy. Curr Cancer Drug Targets.

[7] Turbin DA, Cheang MC, Bajdik CD, Gelmon KA, Yorida E, De Luca A, Nielsen TO, Huntsman DG, Gilks CB (2006). MDM2 protein expression is a negative prognostic marker in breast carcinoma. Mod Pathol.

[8] Phelps M, Darley M, Primrose JN, Blaydes JP (2003). p53-independent activation of the hdm2-P2 promoter through multiple transcription factor response elements results in elevated hdm2 expression in estrogen receptor alpha-positive breast cancer cells. Cancer Res.

[9] Bond GL, Hirshfield KM, Kirchhoff T, Alexe G, Bond EE, Robins H, Bartel F, Taubert H, Wuerl P, Hait W, Toppmeyer D, Offit K, Levine AJ (2006). MDM2 SNP309 accelerates tumor formation in a gender-specific and hormone-dependent manner. Cancer Res.

[10] Hu W, Feng Z, Ma L, Wagner J, Rice JJ, Stolovitzky G, Levine AJ (2007). A single nucleotide polymorphism in the MDM2 gene disrupts the oscillation of p53 and MDM2 levels in cells. Cancer Res.

[11] Brekman A, Singh KE, Polotskaia A, Kundu N, Bargonetti J (2011). A p53-independent role of Mdm2 in estrogen-mediated activation of breast cancer cell proliferation. Breast Cancer Res.

[12] Bond GL, Hu W, Bond EE, Robins H, Lutzker SG, Arva NC, Bargonetti J, Bartel F, Taubert H, Wuerl P, Onel K, Yip L, Hwang SJ (2004). A single nucleotide polymorphism in the MDM2 promoter attenuates the p53 tumor suppressor pathway and accelerates tumor formation in humans. Cell.

[13] Swetzig WM, Wang J, Das GM (2016). Estrogen receptor alpha (ERalpha/ESR1) mediates the p53-independent overexpression of MDM4/MDMX and MDM2 in human breast cancer. Oncotarget.

[14] Bohlman S, Manfredi JJ (2014). p53-independent effects of Mdm2. Subcell Biochem.

[15] Bouska A, Eischen CM (2009). Mdm2 affects genome stability independent of p53. Cancer Res.

[16] Ganguli G, Wasylyk B (2003). p53-independent functions of MDM2. Mol Cancer Res.

[17] Jones SN, Hancock AR, Vogel H, Donehower LA, Bradley A (1998). Overexpression of Mdm2 in mice reveals a p53-independent role for Mdm2 in tumorigenesis. Proc Natl Acad Sci U S A.

[18] Lundgren K, Montes de Oca Luna R, McNeil YB, Emerick EP, Spencer B, Barfield CR, Lozano G, Rosenberg MP, Finlay CA (1997). Targeted expression of Mdm2 uncouples S phase from mitosis and inhibits mammary gland development independent of p53. Genes Dev.

[19] Martin K, Trouche D, Hagemeier C, Sorensen TS, La Thangue NB, Kouzarides T (1995). Stimulation of E2F1/DP1 transcriptional activity by MDM2 oncoprotein. Nature.

[20] Zhang Z, Wang H, Li M, Rayburn ER, Agrawal S, Zhang R (2005). Stabilization of E2F1 protein by MDM2 through the E2F1 ubiquitination pathway. Oncogene.

[21] Sdek P, Ying H, Chang DL, Qiu W, Zheng H, Touitou R, Allday MJ, Xiao ZX (2005). MDM2 promotes proteasome-dependent ubiquitin-independent degradation of retinoblastoma protein. Mol Cell.

[22] Miwa S, Uchida C, Kitagawa K, Hattori T, Oda T, Sugimura H, Yasuda H, Nakamura H, Chida K, Kitagawa M (2006). Mdm2-mediated pRB downregulation is involved in carcinogenesis in a p53-independent manner. Biochem Biophys Res Commun.

[23] Uchida C, Miwa S, Kitagawa K, Hattori T, Isobe T, Otani S, Oda T, Sugimura H, Kamijo T, Ookawa K, Yasuda H, Kitagawa M (2005). Enhanced Mdm2 activity inhibits pRB function via ubiquitin-dependent degradation. EMBO J.

[24] Prall OW, Sarcevic B, Musgrove EA, Watts CK, Sutherland RL (1997). Estrogen-induced activation of Cdk4 and Cdk2 during G1-S phase progression is accompanied by increased cyclin D1 expression and decreased cyclin-dependent kinase inhibitor association with cyclin E-Cdk2. J Biol Chem.

[25] Altucci L, Addeo R, Cicatiello L, Dauvois S, Parker MG, Truss M, Beato M, Sica V, Bresciani F, Weisz A (1996). 17beta-Estradiol induces cyclin D1 gene transcription, p36D1-p34cdk4 complex activation and p105Rb phosphorylation during mitogenic stimulation of G(1)-arrested human breast cancer cells. Oncogene.

[26] Zhang Z, Li M, Wang H, Agrawal S, Zhang R (2003). Antisense therapy targeting MDM2 oncogene in prostate cancer: Effects on proliferation, apoptosis, multiple gene expression, and chemotherapy. Proc Natl Acad Sci U S A.

[27] Bissell MJ, Radisky DC, Rizki A, Weaver VM, Petersen OW (2002). The organizing principle: microenvironmental influences in the normal and malignant breast. Differentiation.

[28] Manni A, Wright C, Buck H (1991). Growth factor involvement in the multihormonal regulation of MCF-7 breast cancer cell growth in soft agar. Breast Cancer Res Treat.

[29] Polotskaia A, Xiao G, Reynoso K, Martin C, Qiu WG, Hendrickson RC, Bargonetti J (2015). Proteome-wide analysis of mutant p53 targets in breast cancer identifies new levels of gain-of-function that influence PARP, PCNA, and MCM4. Proc Natl Acad Sci U S A.

[30] Kenny PA, Lee GY, Myers CA, Neve RM, Semeiks JR, Spellman PT, Lorenz K, Lee EH, Barcellos-Hoff MH, Petersen OW, Gray JW, Bissell MJ (2007). The morphologies of breast cancer cell lines in three-dimensional assays correlate with their profiles of gene expression. Mol Oncol.

[31] Hendzel MJ, Wei Y, Mancini MA, Van Hooser A, Ranalli T, Brinkley BR, Bazett-Jones DP, Allis CD (1997). Mitosis-specific phosphorylation of histone H3 initiates primarily within pericentromeric heterochromatin during G2 and spreads in an ordered fashion coincident with mitotic chromosome condensation. Chromosoma.

[32] Frum RA, Singh S, Vaughan C, Mukhopadhyay ND, Grossman SR, Windle B, Deb S, Deb SP (2014). The human oncoprotein MDM2 induces replication stress eliciting early intra-S-phase checkpoint response and inhibition of DNA replication origin firing. Nucleic Acids Res.

[33] Dolfi SC, Jager AV, Medina DJ, Haffty BG, Yang JM, Hirshfield KM (2014). Fulvestrant treatment alters MDM2 protein turnover and sensitivity of human breast carcinoma cells to chemotherapeutic drugs. Cancer Lett.

[34] Saji S, Okumura N, Eguchi H, Nakashima S, Suzuki A, Toi M, Nozawa Y, Hayashi S (2001). MDM2 enhances the function of estrogen receptor alpha in human breast cancer cells. Biochem Biophys Res Commun.

[35] Clare SE, Shaw PL (2016). “Big Data” for breast cancer: where to look and what you will find. NPJ Breast Cancer.

[36] Dowsett M, Howell R, Salter J, Thomas NM, Thomas EJ (1995). Effects of the pure anti-oestrogen ICI 182780 on oestrogen receptors, progesterone receptors and Ki67 antigen in human endometrium in vivo. Hum Reprod.

[37] Wardell SE, Marks JR, McDonnell DP (2011). The turnover of estrogen receptor alpha by the selective estrogen receptor degrader (SERD) fulvestrant is a saturable process that is not required for antagonist efficacy. Biochem Pharmacol.

[38] Bhattacharya S, Ghosh MK (2014). HAUSP, a novel deubiquitinase for Rb - MDM2 the critical regulator. FEBS J.

[39] Shibata T, Watari K, Izumi H, Kawahara A, Hattori S, Fukumitsu C, Murakami Y, Takahashi R, Toh U, Ito KI, Ohdo S, Tanaka M, Kage M (2017). Breast cancer resistance to antiestrogens is enhanced by increased ER degradation and ERBB2 expression. Cancer Res.

