# Supplementary material for: Estrogen-activated MDM2 disrupts mammary tissue architecture through a p53-independent pathway
Source: Oncotarget. 2017 May 24;8(29):47916–30. doi: 10.18632/oncotarget.18147 (PMC5564615; doi:10.18632/oncotarget.18147)
Supplement: Supplementary file 1 [file oncotarget-08-47916-s001.pdf]

Estrogen-activated MDM2 disrupts mammary tissue architecture through a p53-independent pathway

SUPPLEMENTARY MATERIALS

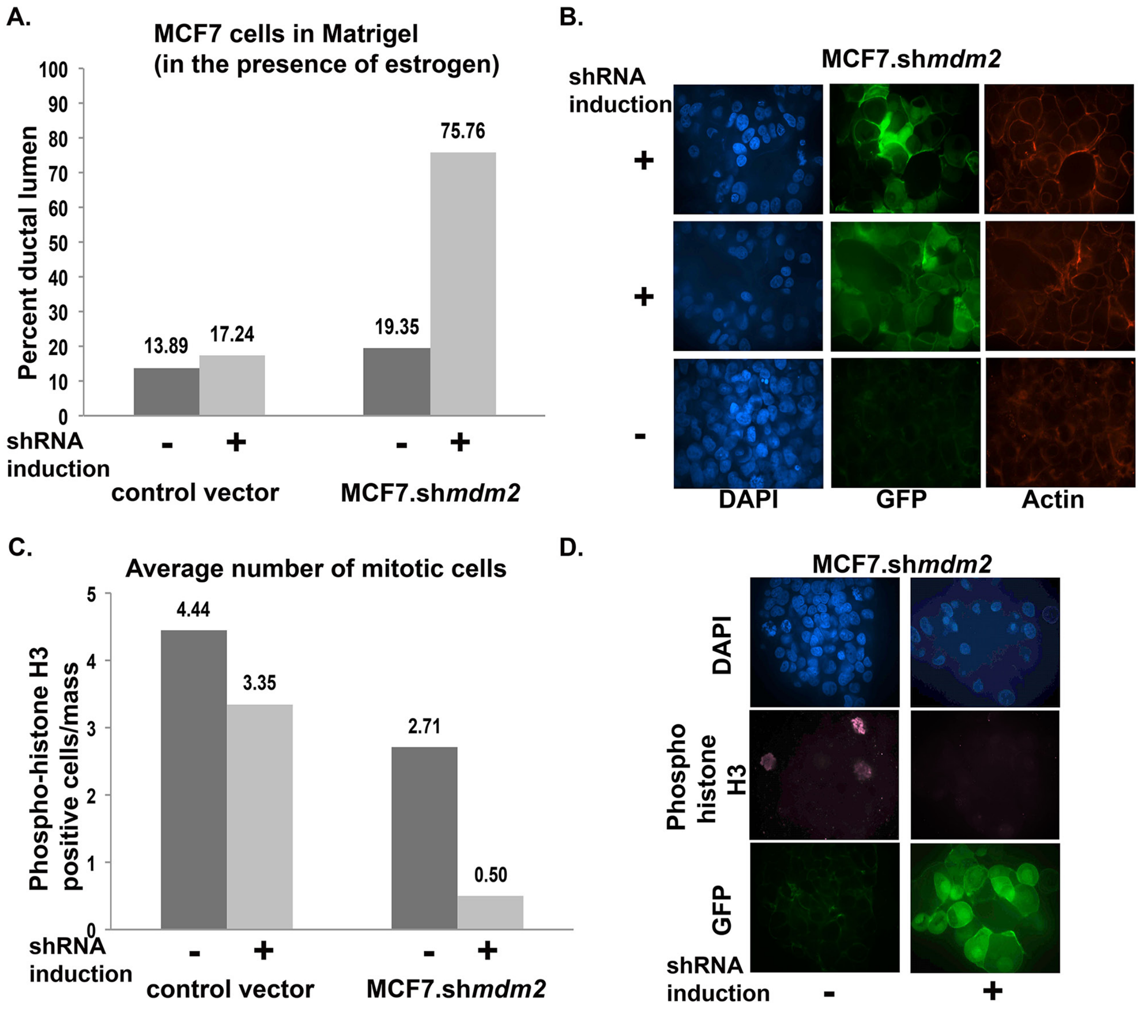

Supplementary Figure 1: MDM2 depletion in ER+ MCF7 cells with wild type p53 reverts mammary architecture and reduces the number of mitotic cells. MCF7 cells (inducible sh*mdm2* clonal and control vector pool) grown in matrigel for 3 weeks in the presence of estrogen and in the presence or absence of 4µg/ml dox, were fixed, permeablilized, blocked, stained with phospho-histoneH3 antibody, F-Actin and mounted with DAPI containing mounting media. Images were taken with confocal microscope. All the masses were scored for the presence or absence of lumen and phospho histone H3 positive cells. Masses with lumen were counted and presented as percentage of total number of masses grown in 3D matrigel. Quantitative analysis of phospho-histone H3 positive cells were made by capturing optical Z-stack sections of masses and dividing the number of positive phospho-histone H3 cells by the total number of masses. Confocal z-stack images of 30 masses in each group were analyzed. **(A)** Graphical representation of percent of masses with ductal lumen. **(B)** A representative image from confocal immunofluorescence microscopy showing a single slice from z-stack of DAPI, GFP and F-Actin of estrogen treated inducible clonal MCF7.sh*mdm2* cells grown in 3D matrigel in the presence and absence of 4µg/ml doxycycline (dox) for 3 weeks. The top and middle rows show ductal lumen in the presence of *mdm2* shRNA expression; the GFP (green) indicates shRNA induction. The third row shows mass structure (disruption of normal mammary glandular architecture) in the absence of shRNA induction. **(C)** Graphical representation showing the number of phospho histone H3 positive cells per mass. **(D)** Representative confocal Z-stack (single slice) image showing DAPI, phospho-histone H3 and GFP in estrogen-treated inducible clonal T47D.sh*mdm2* cells in the presence and absence of 4µg/ml doxycycline.

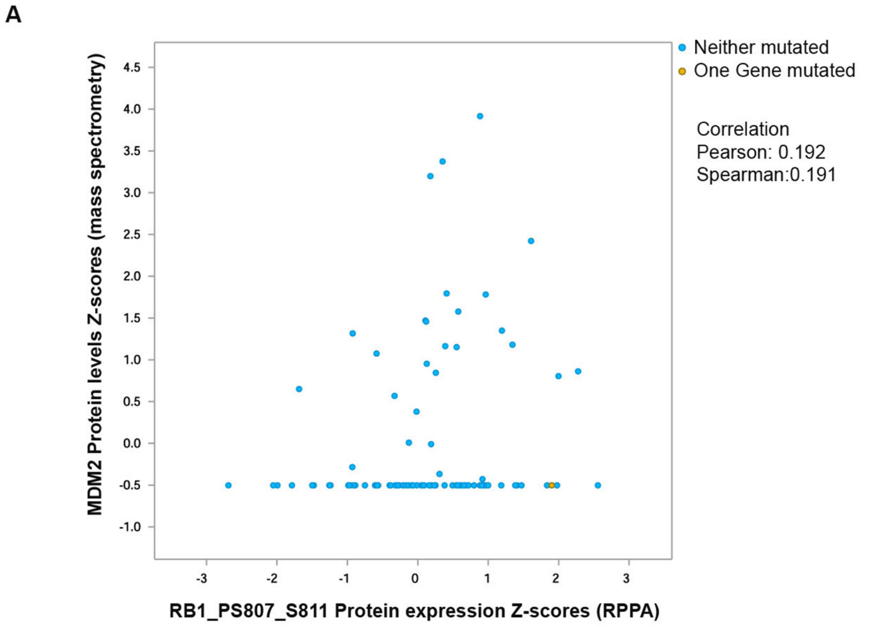

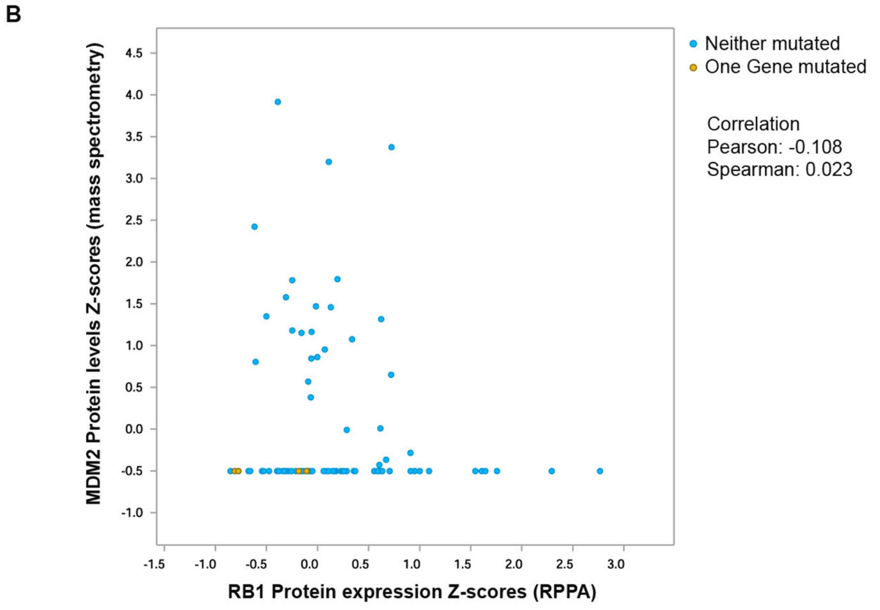

Supplementary Figure 2: Analysis of protein expression correlation of Phospho-RB (RB1_PS807_S811) and MDM2 in breast invasive carcinoma samples from The Cancer Genome Atlas (TCGA) database. Scatter plots of RB1_PS807_S811 and MDM2 **(A)** and RB1 and MDM2 **(B)** protein expression correlation analysis (Pearson's r) using TCGA Breast Invasive Carcinoma Provisional dataset as of March 2017. The protein level (z-score) was determined by mass spectrometry and reverse-phase protein array (RPPA). The plots were generated by cBioPortal, an open-access resource at http://www.cbioportal.org/. Results showed a trend toward to significance positive correlation between RB1_PS807_S811 and MDM2 protein expression (Pearson r = 0.192, p-value=0.0638) and a non-significant negative correlation between RB1 and MDM2 protein expression (Pearson r = -0.108, p-value= 0.2983).

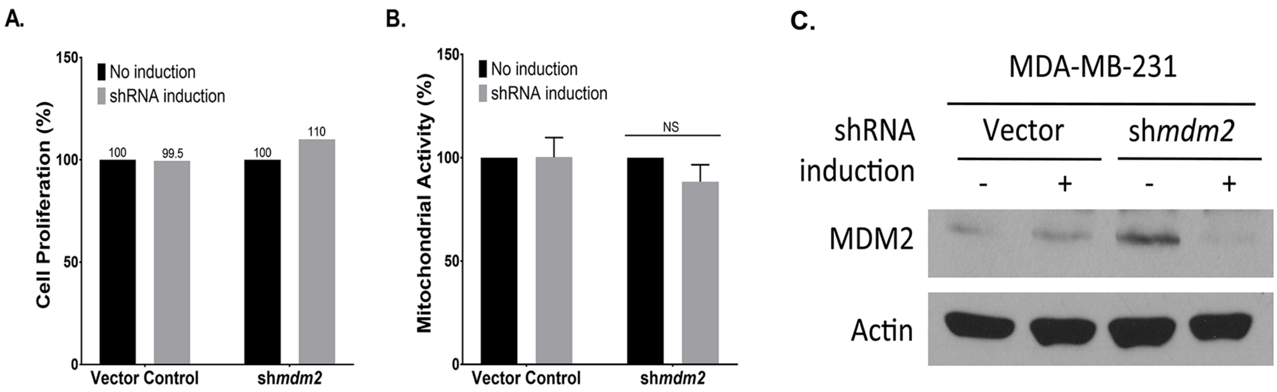

Supplementary Figure 3: MDM2 knockdown in triple negative breast cancer cell line does not alter cell proliferation. Inducible clonal MDA-MB-231 cells with *mdm2* shRNA or vector control were grown with or without 6μg/ml doxycycline (dox) for 5 days to induce shRNA expression. **(A)** Number of cells was determined by cell counting using hemocytometer after 5 days in either presence or absence of shRNA induction. Percentages of cell proliferation were obtained by normalizing to vector control or sh*mdm2* without shRNA induction. Mean values of two biological replicates are shown. **(B)** Mitochondrial activity was determined by MTT assay after 5 days in either the presence or absence of shRNA induction. Percentages of mitochondrial activity were obtained by normalizing to vector control or sh*mdm2* without shRNA induction. Error bars represent standard deviation of three biological replicates. NS: non-significant by Student's t-test. **(C)** A representative image of western blot analysis of MDM2 and Actin protein levels from 50μg whole cell protein extract is shown.
